# Efficacy study of CyberKnife stereotactic radiosurgery combined with CIK cell immunotherapy for advanced refractory lung cancer

**DOI:** 10.3892/etm.2012.818

**Published:** 2012-11-19

**Authors:** YUAN-YUAN WANG, YI-SHAN WANG, TAO LIU, KE YANG, GUI-QING YANG, HAN-CHEN LIU, SHAN-SHAN WANG, JIA-LIN YANG

**Affiliations:** 1 School of the Integration of Traditional and Western Medicine, Binzhou Medical University, Yantai, Shandong 264003;; 2 Center for Tumor Treatment of the People’s Liberation Army 107th Hospital, Yantai, Shandong 264002;; 3 Potenbio Biomedical Technical Company, Shenzhen, Guangdong 518000, P.R. China

**Keywords:** CyberKnife, stereotactic radiosurgery, cytokine-induced killer, immunotherapy, clinical efficacy

## Abstract

CyberKnife (CK), hypofractionated stereotactic radiosurgery, is a preferred option for the treatment of advanced refractory lung cancer which is usually inoperable. Cytokine-induced killer (CIK) cell immunotherapy has a marked radiosensitization effect which aids the elimination of residual tumor cells in distant areas. The main purpose of the present study was to evaluate the clinical efficacy of CK alone and combined with CIK cell therapy for advanced refractory lung cancer. In one year, 22 patients with advanced lung cancer underwent CK therapy at a CyberKnife Center. Of these patients, 11 received CIK cell therapy before or after the CK therapy course. The median prescribed dose in the combined CK and CIK group was 35 Gy (mean, 33.8±5.0 Gy) with a median number of fractions of 5. The median dose for patients who underwent CK alone was 35 Gy (mean, 35.2±6.0 Gy). CIK cell therapy was administered according to the condition of each patient, generally 2 continuous therapeutic sessions in 2 months. The median follow-up period was 3 months. The preliminary curative efficiency rate was 81.82% for patients who underwent CK/CIK and 72.73% for those who received CK alone, according to radiographic re-examination (P>0.05). The median improvement in the Karnofsky scores of the CK/CIK group was 20 (18±10.51) compared with 10 (8.6±11.85) for those who underwent CK alone (P<0.05). The median expression of carcinoembryonic antigen (CEA) before and after treatment was 40.81 and 12.21 ng/ml, respectively, for the CK/CIK group compared with 39.04 and 26.36 ng/ml for CK alone. The median percentage of phenotype expression of the CIK cells (CD3^+^/CD8^+^ and CD3^+^/CD56^+^) in the patients who underwent CK/CIK was recorded as 64.35% (57.08±16.94%) and 15.27% (18.80±7.00%), respectively, prior to transfusion. The preliminary results of the present study suggest that CK combined with CIK cell immunotherapy improved the short-term outcomes of patients for curative efficacy, Karnofsky scores, tumor marker levels and immune status compared with alternative CK treatments, although further studies are required.

## Introduction

Surgical treatment is always advised against for advanced refractory lung tumors (1). Chemotherapy may aid prolonged growth control but may be offered only to a small subpopulation due to the malignant constitution and poor tolerance of the majority of later stage patients (2). The CyberKnife (CK) hypofractionated stereotactic radiosurgery system allows tumor lesions to be safely treated by focused high doses of radiation in a limited number of treatment sessions (3). A number of retrospective studies on the use of CK have suggested that CK may be adopted as a preferred treatment option for advanced refractory tumors (4–6). However, as a focal treatment, CK has limited effects when controlling the metastatic microscopic lesions which often develop in advanced cases. The combination of CK with the cytokine-induced killer (CIK) cell therapeutic strategy may maintain control over local tumors while promoting antitumor activity at distant microscopic lesions and improving the overall immune constitution of the patients. Furthermore, the necrotic tumor lesions may constantly release tumor antigen due to the CK treatment, resulting in the activation of dendritic cells. Dendritic cells are the most powerful antigen-presenting cells in the human body.

CIK cells are the most important approach in adoptive cellular immunotherapy and have demonstrated an attractive augmentation of therapeutic activity against certain types of tumors when combined with chemotherapy (7). However, there are no data concerning the inhibitory effects of CIK cells combined with CK on the development of advanced tumors. In the present study, the clinical efficacies of CK alone and combined with CIK cell therapy were analyzed for the management of advanced refractory lung cancer. The preliminary data are presented in the present study.

## Patients and methods

### Patient characteristics

Between November 2010 and August 2011, 22 patients with advanced refractory lung cancer underwent CK therapy at the Center for Tumor Treatment of the People’s Liberation Army 107th Hospital (Yantai, China). The inclusion criteria included: i) pathological or radiographic confirmation of stage III–IV lung tumors; ii) Karnofsky performance status (KPS) ≥50; iii) life expectancy ≥3 months; iv) hemogram (hemachrome >80 g/l, white blood cell count >3×10^7^ /l), blood urea nitrogen, serum creatinine, alanine aminotransferase (ALT), aspartate aminotransferase (AST) and alkaline phosphatase were close to normal levels; and v) no use of corticosteroids within 6 weeks. All the included patients gave informed consent for detection and treatment.

### Methods

All the recruited patients experienced a 1-week clearance period to recover from the toxicity of previous treatments. CK treatments were planned and delivered in 3 to 8 fractions and the whole treatment was completed in 1 week. Therapeutic doses were determined according to the volume, location and stage of the tumor. The gross tumor volume (GTV) and planned treatment volume (PTV) were measured using CT scans (1.25 mm), MRI, PET-CT or DSA image fusion. To mark the target areas, four patients received 1 to 2 fiducial markers. The overall doses ranged between 24 and 47 Gy and fraction doses were 4.4 to 12 Gy.

CIK cell therapy was administered to patients in the study group prior to or following the CK therapy session. The CIK cell therapy involved obtaining 50 ml peripheral vein blood, then the cytokine induction and expansion of CIK cells *in vitro* and cell transfusion into the patients. Peripheral blood mononuclear cells (PBMCs; 2×10^7^–3×10^7^) were isolated using Ficoll for the CIK cell culture. Certain types of cytokines were used to induce the differentiation, proliferation and amplification of the CIK cells. Mature CIK cells (∼2×10^10^) were collected on the 14th day and infused into saline for cell infusion. The management of CIK cell therapy was dependent on the status of the patients and generally consisted of 2 continuous therapeutic courses (CIK cell transfusions twice for each therapeutic course) in 2 months. The clinical treatment schemes are shown in Table I.

### Assessment of efficacy and statistical analysis

The efficacy was evaluated using radiographic re-examination, Karnofsky scores, tumor markers and CIK cell phenotype detection. All comparisons were performed between the control group (CK alone) and study group (CK/CIK) in the present study. The Chi-square test was used to compare the preliminary curative efficiency rate between the two samples. A comparison of the Karnofsky scores was performed using the Wilcoxon Rank test. The t-test was used to compare the expression levels of carcinoembryonic antigen (CEA). P<0.05 was considered to indicate statistically significant differences. The SPSS 13.0 software was used for statistical analysis.

## Results

Between November 2010 and August 2011, 22 patients (19 male and 3 female; median age, 61 years) with advanced refractory lung cancer underwent CK therapy at the Center for Tumor Treatment of People’s Liberation Army 107th Hospital. The number of tumor lesions for each patient ranged from one to two. The largest tumor lesion was 15×9.5×18 cm while the smallest was 1.4×1.4×1 cm. A total of six cases also had moderate to severe hydrothorax.

All the patients fulfilled the inclusion criteria and 11 patients were offered the concomitant CK/CIK treatment. The median CK doses for patients who underwent CK/CIK were 35 Gy (mean, 33.8±5.0 Gy) with a median prescription isodose line of 74% (range, 69–81%). The median dose for patients who underwent CK alone was 35 Gy (mean, 35.2±6.0 Gy) at 75% isodose line (range, 66–82%).

Of the patients who underwent CIK cell therapy, two received four CIK transfusions and nine patients received two. No opportunistic infections or capillary leak syndrome occurred during the CIK cell infusions. Acute side effects included fever and allergies. Two patients suffered from fever within 1–3 h following the transfusion with temperatures increasing from 37.6 to 40.0°C. The majority of patients did not require special treatment and physical strategy or anti-inflammatory drugs were used only when temperature was >39°C, which rarely occurred. One patient exhibited allergy symptoms for 30 min following the CIK cell transfusion, although the transient chills and fever disappeared soon after the patient was treated with an anti-allergen.

The median follow-up period was 3 months. Preliminary clinical data are shown in Table II.

## Discussion

CK hypofractionated stereotactic radiosurgery has emerged as a promising treatment for early stage lung cancer (3). In patients with advanced lung cancer, CK has also been demonstrated to be a safe and highly effective therapy with only slight adverse reactions (4–6).

The majority of cases of lung cancer are discovered at advanced stages, meaning that the disease has become the leading cause of cancer-related mortality worldwide (8). The current standard treatments for patients with advanced lung cancer are palliative surgical lobectomy, radiotherapy or palliative chemotherapy (9). A large number of patients are deemed to be medically inoperable or chemotherapy intolerant due to poor physical condition. For these patients, conventional radiotherapy is one of the preferred treatment options (10). However, the cumulative doses are limited by considerations of protecting the surrounding normal tissues, leading to a low local control ratio and high recurrence rate (11). CK is often used in the treatment of lung tumors due to an additional component called the Synchrony Respiratory Tracking System.

An increasing number of studies have emerged, demonstrating the promise of CK for treating lung cancer (12–16). Previous results demonstrated that CK is an effective palliative treatment option for advanced lung cancer but its inhibition of the recurrence rate is not ideal (17). Dose escalation alone is unlikely to enhance the inhibitory ratio of recurrence and novel approaches, such as combining CK use with other therapeutic strategies, should be investigated thoroughly (18). A number of analyses of clinical research revealed the main predictor of local control by CK to be tumor volume (19). The larger the tumor volume, the lower the tumor local control rate which may be associated with the poor overall condition of the patients. In the present study, CK therapy was combined with CIK cell therapy, aiming to significantly enhance patient immunity, eliminate residual tumor cells and improve the curative effects for advanced lung cancer.

Immunotherapy has evolved over the past 20 years to become an increasingly attractive approach for the treatment of human cancers (20). Adoptive cellular immunotherapy, which eliminates cancer cells and stimulates patient immunity through the transfer of *in vitro* amplified and activated immune cells, is proving to be an effective strategy for cancer therapy (21). CIK cells, a unique population of cytotoxic T cell sharing the functional and phenotypic properties of T and natural killer cells, are considered to be the most promising adoptive cellular therapeutic strategy (22). In contrast to other immune cells, CIK cells have several advantages: i) CIK cells are readily induced *in vitro* and are easy to amplify from the PBMCs of cancer patients to produce a large number of effective cells (23); ii) CIK cells have stronger antitumor ability than lymphokine-activated killer (LAK) cells (24); iii) the cytotoxicity of CIK cells is not major histocompatibility complex-restricted and CIK cells do not induce graft versus host reaction (GvHR) (24,25); iv) as the final effector cells, CIKs are capable of killing cancer cells directly; and v) CIK cells remain effective against MDR, FasL-positive malignant cells and possibly anti-radiation malignant cells (26). In the present study, the technique of CIK cell culture developed by Potenbio Biomedical Technical Company in Shenzhen was adopted. Quality control tests of the CIK cells were performed during culture and prior to transfusion. The detection of cell activity using FACS and cell counts confirmed that survival ratios were over 99.5% and 2×10^10^ CIK cells were infused into patients.

In the present study, the percentage of CD3^+^/CD56^+^ dual positive cells was approximately 15%, which appeared to be lower than certain previous reports (40% is the highest achieved at present). All cells in the final transfusion products were also analyzed. The percentage of CD3^+^ cells was 98.5%, indicating that the majority of the cells were T cells. Although some cells were CD56-negative, the large quantity of T lymphocytes were able to participate in the process of secreting multiple cytokines, including IL2, TNF-α and γ-INF. These cytokines are capable of increasing the anticancer ability of the human body. The absolute count of CD3^+^/CD56^+^ dual positive cells was not low due to the high final number of CIK cells. The final number of CD3^+^/CD56^+^ dual positive cells in the present study was a maximum of 3×10^9^, which was not lower than previously published studies.

There are numerous studies concerning the use of CK or CIK cell therapy against advanced lung cancer but this is the first study to address CK combined with CIK immunotherapy. The preliminary results revealed that the combination of CK and CIK immunotherapy may counteract malignant cells synergetically and be beneficial to patients with advanced refractory lung cancer.

## Figures and Tables

**Table I.** Clinical treatment schemes of all patients.

No.	Volume (cm)	Fractions	Total dose (Gy)	Biological equivalent doses (Gy)	CIK cell infusions
1–1	15x9.5x18	8	35	50	0
1–2	5.7x3x4.4	5	30	47	0
1–3	4x4x4	5	30	47	0
1–4	6x5x8	5	35	58	0
1–5	4.5x4.5x5, 3x3.5x2	5	35	58	0
1–6	4x3x3	5	35	58	0
1–7	3x3x1.6	4	25	39	0
1–8	2.5x2.5x2.5	6	47	84	0
1–9	6x6x8	5	40	72	0
1–10	3x2x3, 2x2x3	5	40	72	0
1–11	7x7x7	5	35	58	0
2-1	7x7x7	5	35	58	4
2–2	8x8x3	6	42	73	4
2–3	10x7x10	6	40	66	2
2–4	3x6x6, 4x3x6	5	35	58	2
2–5	3.5x4x3	3	36	78	2
2–6	7.8x8.2x6	5	35	58	2
2–7	8x7x8.5, 1.4x2.5x4.5	5	33	55	2
2–8	5x4x2.5	5	29	45	2
2–9	10x10x10	5	30	47	2
2–10	3.2x2.7x4, 1.4x1.4x1	5	24	35	2
2–11	8.0x2.3x4.5	5	33	55	2

CIK, cytokine-induced killer.

**Table II.** Clinical results of CK only or CK/CIK therapy.

Characteristic	CK	CK/CIK cell therapy	P-value
Efficacy assessment (WHO; %)	72.73	81.82	0.118
KPS, mean ± SD			
Before	65.00±10.00	67.73±9.05	0.029
After	73.64±11.20	86.36±11.64	
Tumor marker (CEA), mean ± SD			
Before	185.05±338.41	181.00±305.56	0.045
After	102.27±194.39	45.10±73.27	
Phenotype of CIK cells before transfusion, mean ± SD (%)			
CD3^+^/CD8^+^ (d12)	-	57.08±16.94	-
CD3^+^/CD56^+^ (d12)	-	14.80±7.00	-

CK, CyberKnife; CIK, cytokine-induced killer; KPS, Karnofsky performance status; CEA, carcinoembryonic antigen.
